# Evaluation of plaque removal effectiveness of a microcurrent-emitting toothbrush among patients with fixed orthodontic appliances

**DOI:** 10.6026/973206300212758

**Published:** 2025-08-31

**Authors:** Dhananjay Rathod, Saakshi Rane, Mayur Kumar Soni3, Vishal Patel, Nivedita Sahoo, Ananya Bhargava, Miral Mehta

**Affiliations:** 1 Department of Dentistry, Sheikh Bhikhari Medical College, Hazaribagh, Jharkhand; 2 Department of Dentistry, Chatrapati Sambhaji Maharaj Government Medical College and Hospital, Sadar Bazar, Satara 415001; 3 Department of Dentistry, Netaji Subhash Chandra Bose Medical College and Hospital, Jabalpur, Madhya Pradesh, India; 4 Department of Orthodontics and Dentofacial Orthopaedics, Faculty of Dental Science, Dharmsinh Desai University, Nadiad; 5 Department of Orthodontics and Dentofacial Orthopaedics, Kalinga Institute of Dental Sciences, KIIT (Deemed to be University),Bhubaneswar, Odisha, India; 6 Department of Dentistry, Ruxmaniben Deepchand Gardi Medical College, Ujjain, Madhya Pradesh, India; 7 Department of Pediatric and Preventive Dentistry, Karnavati School of Dentistry, Karnavati University, Gandhinagar, Gujarat, India

**Keywords:** Micro current toothbrush, orthodontic treatment, plaque removal, bioelectric effect, oral hygiene

## Abstract

The effect of a microcurrent-emitting toothbrush (MCT) versus a conventional toothbrush (CT) in 40 orthodontic patients is of interest. The MCT significantly reduced plaque levels (mean difference: 8.2%, p<0.001) and improved gingival health (p=0.003), especially near brackets and interproximal areas. Patient satisfaction was also higher for MCTs (p=0.008). Thus, we show the use of MCTs for enhanced oral hygiene during fixed orthodontic treatment.

## Background:

Maintaining optimal oral hygiene during orthodontic treatment presents a significant challenge for patients and clinicians a like [1]. The presence of fixed orthodontic appliances creates numerous plaque-retention sites, markedly increasing the risk of enamel demineralization, white-spot lesions and gingivitis [2]. Studies have shown that orthodontic patients experience up to 65 - 67 % deterioration in oral-hygiene status shortly after appliance placement, with approximately 60 % displaying poor oral hygiene during treatment [3]. The bracket-tooth interface remains a main reservoir for plaque accumulation even after brushing, thereby elevating the likelihood of enamel demineralization [4]. Dental plaque around orthodontic appliances can enlarge the total microbial population and shift the ecosystem, leading to chronic infections such as periodontitis [5]. Because fixed orthodontic therapy commonly exceeds two years, sustained plaque control is essential for favorable outcomes and oral-health maintenance [6]. Conventional mechanical plaque-removal methods often fall short in orthodontic patients, as the intricate configuration of brackets and wires hinders thorough cleaning [7]. Although various specialized brushes and interdental aids exist, compliance is low-about 78 % of patients do not follow recommended regimens despite repeated instruction [8]. Microcurrent-emitting toothbrushes (MCTs) have recently been introduced; they deliver low-intensity electric currents (100 - 500 μA) that disrupt biofilm structure through electrostatic forces, potentially enhancing plaque removal beyond mechanical action. The bioelectric effect weakens biofilm integrity, alters bacterial metabolism, and-through combined AC/DC stimulation-loosens the matrix for easier elimination [9]. Micro current therapy also appears to boost adenosine triphosphate (ATP) production in gingival tissues, which may lessen inflammation and support healing. Ion migration toward the cathode is thought to stimulate mitochondrial ATP synthesis, offering anti-inflammatory benefits that complement plaque removal [10]. While several studies have evaluated MCTs in general populations, data specific to orthodontic patients remain scarce. A recent trial reported a 9.43 % plaque-index reduction with MCTs versus 1.42 % with conventional brushes in fixed-appliance wearers [9]. However, detailed assessment of plaque removal on all tooth surfaces-especially interproximal and bracket-adjacent areas-remains limited [2]. Therefore, it is of interest to investigate the plaque-removal efficiency of an MCT compared with a conventional toothbrush in patients undergoing fixed orthodontic treatment, with particular attention to difficult-to-access sites. We hypothesized that the MCT would outperform the conventional brush, especially around brackets and in interproximal regions.

## Materials and Methods:

This study employed a randomized, controlled, crossover design and was conducted between January 2024 and April 2024. The sample size was calculated using G*Power software, assuming a medium effect size, 80% power, and a significance level of 5%. Based on this, a minimum of 34 participants was required, and with an additional 15% allowance for potential dropouts, the final sample size was determined to be 40 participants. Patients undergoing fixed orthodontic treatment were recruited based on specific inclusion criteria, which included individuals aged between 15 and 30 years, undergoing treatment with brackets on at least 20 teeth, having completed at least three months of treatment before enrollment, and being in good general health. Exclusion criteria involved systemic conditions or medications affecting oral health, pregnancy or lactation, severe periodontal disease, inability to independently maintain oral hygiene, or known allergies to toothpaste ingredients. Written informed consent was obtained from all participants or their legal guardians in the case of minors. Participants were randomly allocated to two groups using computer-generated random numbers. Group A received the microcurrent-emitting toothbrush (MCT) during the first phase, followed by the conventional toothbrush (CT), while Group B followed the reverse sequence. Allocation was concealed through sequentially numbered, opaque, sealed envelopes. Although complete blinding was not feasible due to visible differences between the toothbrushes, outcome assessors and statisticians remained blinded to group assignments. The interventions involved two types of toothbrushes. The MCT used was a commercially available product (ProxyWave®) emitting a microcurrent of approximately 100 μA through electrodes on the brush head, combining alternating and direct current to disrupt plaque biofilm. The CT was a standard manual toothbrush with a similar bristle configuration but lacking any microcurrent technology. All participants were provided with the same fluoride toothpaste and standardized instructions for brushing using the modified Bass technique twice daily for two minutes. The crossover design included two intervention periods of four weeks each, separated by a one-week washout period during which participants used a standard manual toothbrush provided by the researchers to minimize any carryover effects. Clinical assessments were conducted at four time points: baseline (T0), after the first intervention (T1), post-washout (T2), and after the second intervention (T3). Two calibrated examiners performed evaluations for Plaque Index (PI) using Attin's orthodontic plaque index, Gingival Index (GI) using the Löe and Silness method, and patient satisfaction using a visual analog scale (VAS) assessing cleaning efficacy, comfort, freshness, and overall satisfaction. Calibration sessions before the study ensured high inter-examiner reliability with an intraclass correlation coefficient exceeding 0.85 for both indices. Statistical analysis was carried out using SPSS version 27.0. Data normality was assessed using the Shapiro-Wilk test. Descriptive statistics included means, standard deviations, and percentages. The effectiveness of the two toothbrushes was compared using paired t-tests for normally distributed data and Wilcoxon signed-rank tests for non-normal data. Repeated measures ANOVA were applied to evaluate changes in PI and GI over time, with Bonferroni correction for multiple comparisons. Carryover effects were examined through independent t-tests comparing summed outcomes between sequences, while period effects were evaluated by comparing outcome differences regardless of the sequence. Patient satisfaction scores were analyzed using paired t-tests, and subgroup analyses based on age, gender, and duration of orthodontic treatment were performed using independent t-tests or Mann-Whitney U tests where appropriate. A significance level of p < 0.05 was maintained throughout all statistical analyses.

## Results:

Of the 40 participants enrolled, 37 completed the study (92.5% completion rate). Three participants withdrew due to personal reasons unrelated to the interventions. The final analysis included 37 participants (19 in Group A and 18 in Group B). Demographic and baseline characteristics are presented in Table 1 (see PDF). No significant differences were observed in baseline characteristics between the two groups, indicating successful randomization. Both toothbrushes demonstrated a reduction in plaque scores from baseline, but the MCT showed significantly greater plaque reduction compared to the CT (Table 2 - see PDF). The mean percentage reduction in PI was 32.6% for the MCT compared to 24.4% for the CT (p<0.001). Surface-specific analysis revealed that the MCT was particularly effective in removing plaque from interproximal (mesial and distal) surfaces and areas gingival to the bracket, with statistically significant differences compared to the CT (p<0.001). However, the difference in plaque reduction for surfaces incisal/occlusal to the bracket was not statistically significant (p=0.062). Analysis of carryover and period effects showed no significant influence on the outcomes (p=0.783 and p=0.692, respectively), validating the crossover design. Both toothbrushes resulted in improvement in gingival health, but the MCT demonstrated significantly greater reduction in GI compared to the CT (Table 3 - see PDF). The MCT showed significantly greater improvement in gingival health for interproximal (mesial and distal) surfaces compared to the CT (p<0.01). However, differences in GI reduction for buccal and lingual surfaces were not statistically significant (p>0.05). Patient satisfaction scores were significantly higher for the MCT compared to the CT for cleaning efficacy, freshness sensation, and overall satisfaction (p<0.05). However, there was no significant difference in comfort scores between the two toothbrushes (Table 4 - see PDF). Subgroup analysis based on age, gender, and duration of orthodontic treatment revealed no significant differences in the effectiveness of either toothbrush (p>0.05 for all comparisons), suggesting that the observed benefits of the MCT were consistent across different patient demographics and treatment stages. No adverse events related to either toothbrush were reported during the study period.

## Discussion:

This randomized controlled crossover trial demonstrated that a microcurrent-emitting toothbrush (MCT) provides significantly greater plaque removal efficiency compared to a conventional toothbrush (CT) in patients undergoing fixed orthodontic treatment. The MCT showed an 8.2% greater reduction in plaque index and a 7.9% greater improvement in gingival health compared to the CT, with particularly pronounced benefits in difficult-to-access areas around orthodontic brackets. The superior plaque removal efficiency of the MCT can be attributed to its bioelectric effect, which disrupts the structural integrity of dental biofilms through electrostatic forces [2]. This mechanism complements the mechanical cleaning action of bristles, potentially offering advantages in areas where mechanical cleaning alone is insufficient, such as interproximal spaces and around orthodontic brackets [3]. Our findings align with recent research by Kim *et al.* who reported that MCTs demonstrated a 9.43% decrease in plaque index compared to a 1.42% decrease with conventional toothbrushes in orthodontic patients [1]. The surface-specific analysis revealed that the MCT was particularly effective in removing plaque from interproximal surfaces and areas gingival to the bracket, which is typically the most challenging to clean in orthodontic patients [5]. The bioelectric effect may be especially beneficial in these areas by weakening the biofilm structure, making it more susceptible to removal even with minimal mechanical contact [7]. The improvement in gingival health observed with the MCT may be attributed not only to enhanced plaque removal but also to the potential anti-inflammatory effects of microcurrent therapy [8]. Research has shown that microcurrents can increase adenosine triphosphate (ATP) production, which may help reduce inflammation and promote tissue regeneration [9]. This dual mechanism-enhanced plaque removal and anti-inflammatory effects-may explain the significant improvement in gingival health, particularly in interproximal areas [10]. Patient satisfaction scores were significantly higher for the MCT in terms of cleaning efficacy, freshness sensation and overall satisfaction, which may positively influence compliance with oral hygiene practices [11]. This is particularly important for orthodontic patients, who often struggle with maintaining adequate oral hygiene due to the challenges posed by fixed appliances [9]. The perception of enhanced cleaning efficacy may motivate patients to adhere to recommended brushing routines, potentially improving long-term oral health outcomes during orthodontic treatment. This finding is consistent with previous research by Lee *et al.* who observed that microcurrent technology showed enhanced efficacy in interproximal areas where toothbrush bristles cannot reach effectively [12]. The crossover design employed in this study allowed each participant to serve as their control, minimizing the influence of individual variations in oral hygiene practices and susceptibility to plaque formation [13, 14]. The absence of significant carryover and period effects validates the study design and strengthens the reliability of our findings. The high completion rate (92.5%) and absence of adverse events suggest that MCTs are well-tolerated and acceptable to orthodontic patients [15]. Our findings have important clinical implications for orthodontic practice. Given the high prevalence of poor oral hygiene among orthodontic patients (reported to be as high as 60-67%), the use of MCTs could be a valuable addition to oral hygiene protocols. The enhanced plaque removal in difficult-to-access areas may help reduce the risk of enamel demineralization, white spot lesions, and gingivitis, which are common complications of orthodontic treatment. Several limitations of this study should be acknowledged. First, the relatively short intervention periods (4 weeks each) may not reflect the long-term effects of MCT use throughout the entire duration of orthodontic treatment. Second, complete blinding was not possible due to the visible differences between the toothbrushes, which may have introduced some bias in patient-reported outcomes. Third, while we standardized toothpaste use and brushing technique, variations in individual brushing habits at home could not be completely controlled. Future studies should consider incorporating objective measures of brushing behavior, such as electronic monitoring systems, to address this limitation.

## Conclusion:

Microcurrent-emitting toothbrushes significantly enhance plaque removal and gingival health in orthodontic patients, especially around brackets. Their dual action-mechanical cleaning and bioelectric disruption-offers advantages over conventional brushes. Thus, we show their potential as an effective oral hygiene aid during fixed orthodontic treatment.
